# An Ultra-Low Power Turning Angle Based Biomedical Signal Compression Engine with Adaptive Threshold Tuning

**DOI:** 10.3390/s17081809

**Published:** 2017-08-06

**Authors:** Jun Zhou, Chao Wang

**Affiliations:** 1 School of Communication and Information Engineering, University of Electronic Science and Technology of China, Chengdu 611731, China; jun.zhou.sg@ieee.org; 2 Department of Engineering Product Development, Singapore University of Technology and Design, Singapore 487372, Singapore

**Keywords:** ultra-low power, biomedical signal compression, turning angle, healthcare monitoring

## Abstract

Intelligent sensing is drastically changing our everyday life including healthcare by biomedical signal monitoring, collection, and analytics. However, long-term healthcare monitoring generates tremendous data volume and demands significant wireless transmission power, which imposes a big challenge for wearable healthcare sensors usually powered by batteries. Efficient compression engine design to reduce wireless transmission data rate with ultra-low power consumption is essential for wearable miniaturized healthcare sensor systems. This paper presents an ultra-low power biomedical signal compression engine for healthcare data sensing and analytics in the era of big data and sensor intelligence. It extracts the feature points of the biomedical signal by window-based turning angle detection. The proposed approach has low complexity and thus low power consumption while achieving a large compression ratio (CR) and good quality of reconstructed signal. Near-threshold design technique is adopted to further reduce the power consumption on the circuit level. Besides, the angle threshold for compression can be adaptively tuned according to the error between the original signal and reconstructed signal to address the variation of signal characteristics from person to person or from channel to channel to meet the required signal quality with optimal CR. For demonstration, the proposed biomedical compression engine has been used and evaluated for ECG compression. It achieves an average (CR) of 71.08% and percentage root-mean-square difference (PRD) of 5.87% while consuming only 39 nW. Compared to several state-of-the-art ECG compression engines, the proposed design has significantly lower power consumption while achieving similar CRD and PRD, making it suitable for long-term wearable miniaturized sensor systems to sense and collect healthcare data for remote data analytics.

## 1. Introduction

Wearable devices such as smart watches and ECG patches are drastically changing our everyday life by data collection, processing, and analytics [1,2,3,4,5,6,7,8,9,10,11]. For example, wearable healthcare monitoring devices based on body area network require hundreds of sensors to collect and transmit healthcare data in real time for signal processing and data analysis in a sensor node, a base station or a cloud data center [3,7,8]. However, long-term healthcare monitoring generates tremendous data volume and demands significant wireless transmission power and latency, which impose a big design challenge for wearable healthcare sensors powered by batteries [9]. For instance, a typical wireless EEG (electroencephalogram) recording system with 20 channels and a 4 kHz sampling rate approximately consumes more than 3 kJ by transmitting 10 gigbytes of data every day for chronic seizure detection [10]. Significant efforts on both algorithm and hardware design have been made to reduce the power consumption of healthcare sensors by implementing low-power signal processing and data pattern recognition in sensor nodes [1,10,11,12,13]. However, due to limited computing and storage resources and the ever-increasingly complexity of data analytic applications, part of the data processing tasks (e.g., high-level analysis) still needs to be offloaded to the edge or cloud side [4,6,14]. Furthermore, to provide reliable diagnosis and medical service, doctors may still need the original data. To reduce power consumption, a common solution is to compress the data locally to reduce required bandwidth and energy consumption before data transmission to the base station and cloud sides. Efficient compression engine design to reduce the wireless transmission data rate with ultra-low power consumption is essential for wearable healthcare sensor systems in the era of big data and sensor intelligence.

Biomedical signal recording is required in many wearable health monitoring and analytic applications such as ECG (electrocardiography), EEG, blood pressure monitoring, etc. These applications often involve capturing the biomedical signals and wirelessly transmitting them to a remote site for diagnosis. For example, state-of-the-art wireless ECG monitoring devices capture the ECG signal and wirelessly transmit the signal to a computer or mobile phone for display and diagnosis [15,16,17,18,19]. The ECG data can be further transmitted and uploaded to a cloud-side data center for more advanced data analysis in real time. Such devices—including the wearable sensor nodes, mobile phones, and tablet computers—are usually powered by batteries, therefore the system power consumption is strictly constrained. Previous research has found that the power consumption of wireless transmission dominates the overall power consumption of the system [17,18]. As the required signal precision or monitoring time increases, the amount of data for wireless transmission increases significantly, resulting in shorter battery life. If the amount of transmitted data can be reduced, the system power consumption can be lowered proportionally. The transmission bandwidth and data storage can also be minimized. 

In the past, many techniques have been proposed to reduce the amount of transmitted data [15,16,17,18,19,20,21,22,23,24,25]. They can be categorized into time-domain compression, transformed-domain compression, and compressed sensing. 

The time-domain compression methods are usually based on amplitude or slope detection. For example, in the AZTEC method [15], only signal with amplitude larger than a predefined threshold is recorded. This achieves data reduction with large signal distortion. In the turning point (TP) technique [19], two slopes are calculated from three neighboring samples and the sample which gives a larger slope is stored while the other is discarded. The drawback of this method is limited compression ratio (CR) as only half samples are dropped. Another time-domain compression technique is adaptive sampling [16,17], where the sampling rate is varied according to the slope. A high sampling rate is applied for a large slope while a low sampling rate is applied for a small slope. This approach can achieve a large CR. However, using slope to determine the sampling rate is not always valid. For example, in ECG signal the PT waves has a smaller slope than QRS waves. However, a low sampling rate will cause missing or inaccurate detection of PT peaks, which are equivalently important as the QRS peaks in the ECG diagnosis.

The compression can also be performed in the transformed domains. The motivation behind transformed-domain compression is that the signal energy is clustered within a small portion of samples and most of the transformed samples can be discarded without losing much quality during signal reconstruction, since they are small in magnitude. Some well-known transformations adopted for data reduction include discrete wavelet transform (DWT) [20,21,22] and discrete cosine transform (DCT) [23]. These methods can achieve large data reduction with acceptable signal quality. However, they often have high complexity as both transformation and encoding are required, resulting in large power consumption in signal processing, which decreases the battery life. In addition, the final diagnosis is usually done in the time domain, so it is not straightforward to determine good criteria for the compression in the transformed domain.

Compressed sensing (CS) is another data compression methodology which can help reduce data transmission power with a large compression ratio [24,25]. In a basic CS framework, the compressed signal is obtained through multiplication of the original signal and a measurement matrix. Although compressed sensing is able to reduce power consumption while achieving a competitive compression ratio, the quality of the signal is compromised. In addition, compared to other data compression methods, more challenges will be encountered during the data recovery, especially when the input signal is not sparse. 

In this paper, we proposed a turning angle based biomedical signal compression engine with adaptive threshold tuning for healthcare data sensing and analytics in the era of big data and sensor intelligence. The proposed compression engine consumes extremely low power and is able to achieve significant data reduction while maintaining acceptable signal quality for diagnosis. The rest of the paper is organized as follows: in Section 2, the details of the proposed biomedical signal compression engine are presented. In Section 3, the post-layout simulation results are shown and the proposed design is compared with the state-of-the-art designs. In Section 4, the conclusions are drawn.

## 2. Proposed Ultra-Low Power Turning Angle Based Compression Engine with Adaptive Tuning 

**Figure 1:**
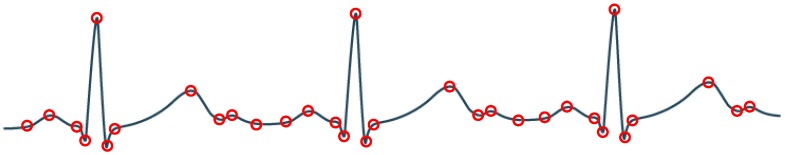
Concept of proposed feature point extraction based on turning angle detection for an ECG signal.

For biomedical compression engines aimed at wearable health monitoring applications (e.g., battery-supplied wireless monitoring devices), a design focus is to achieve a large CR with acceptable signal quality so that the power consumption in wireless transmission can be greatly reduced. Another design focus is to minimize power consumption of the compression engine itself. As the recorded signal is finally used for diagnosis, one way to achieve a large CR with acceptable signal quality is to perform compression focused on the signal features related to diagnosis. By analyzing different types of biomedical signals—such as ECG, EEG, and neural signal—we have observed that the diagnostic information is usually contained in the abrupt turns of the waveform. For example, the QRS/P/T waves in ECG signal, the spike-and-wave in EEG signal, and the action potential in neural signal. Based on this observation, we can extract the feature points containing potential diagnostic information by detecting turning angles larger than a threshold, as shown in Figure 1. This will achieve significant data reduction while maintaining sufficient information for diagnosis. Another advantage of the turning angle based compression is that it has very low complexity as the operation is simple and no transform is required. So the power consumption is low, which is suitable for battery-powered long term monitoring.

Besides performance and power, another consideration for a biomedical signal compression engine is that the signal characteristic varies from person to person or from channel to channel (e.g., multi-channel ECG/EEG). Therefore, a fixed threshold for detection may lead to feature loss or a low CR. To address this issue, we propose to adaptively tune the detection threshold according to the computed error between the original signal and reconstructed signal so that an optimal CR is achieved while meeting the required signal quality.

**Figure 2:**
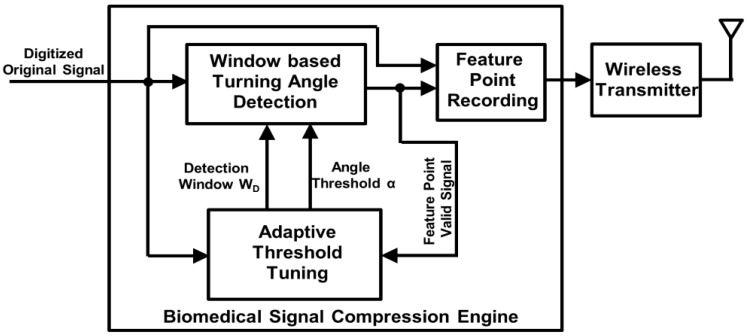
Architecture of the proposed biomedical signal compression engine.

The architecture of the proposed compression engine is shown in Figure 2. We assume that the input signal has been smoothed and digitized by analog frontend and ADC (analogue-to-digital converter). A window-based turning angle detection module is designed to extract the feature points for signal compression. After the extraction, the values of the feature points and their interval will be recorded and wirelessly transmitted for later signal reconstruction and diagnosis. An adaptive threshold tuning module is designed to adjust the turning angle threshold according to the computed error between the original input signal and reconstructed signal using the feature points. The details of the proposed biomedical processing engine are described in the following subsections.

### 2.1. Feature Point Extraction Using Window-Based Turning Angle Detection

**Figure 3:**
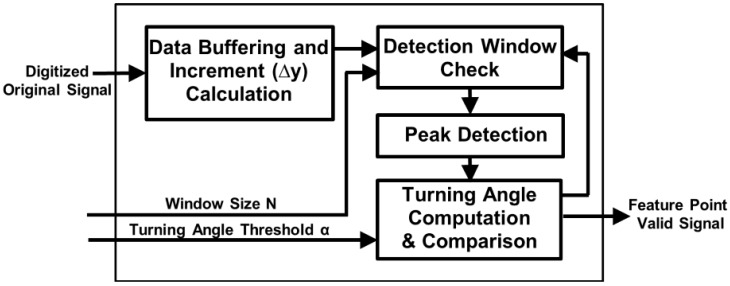
Window-based turning angle detection module.

**Figure 4:**
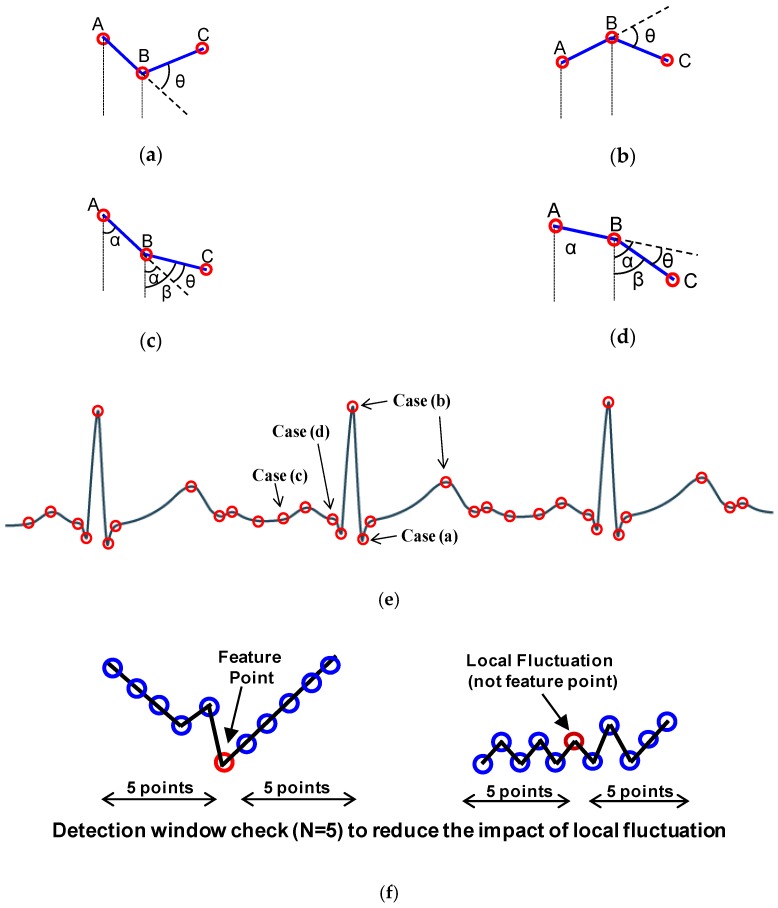
Four cases in turning angle detection. (**a**) Case I; (**b**) Case II; (**c**) Case III; (**d**) Case IV; (**e**) the four cases in an ECG signal example; (**f**) detection window check.

The proposed window-based turning angle detection module is shown in Figure 3. The detection is performed based on three consecutive samples. Take the three samples (A, B, C) with different turning patterns depicted in Figure 4 as an illustrative example. First, peak detection will be performed on the samples. For cases (a) and (b), the slope of AB and BC has different signs, indicating that B is a local peak. In these cases, we will directly record B as a feature point without turning angle computation. For the cases (c) and (d), B is not a local peak. We will first perform turning angle computation to see if the turning angle θ from AB to BC is significant. If the turning angle is larger than the predefined angle threshold, we will record point B as a feature point.

The derivation of turning angle is described in Equations (1)–(3) where the turning angle θ represents the angle between the extended line of AB and BC. If the computed tanθ is larger than tanα, where α is the predefined threshold for turning angle, or as Equation (3), the point B will be recorded as a feature point. In addition to the values of the feature points, the interval (i.e., number of points) between two neighboring feature points will also be recorded for future signal reconstruction.

(1) $$\begin{array}{l} \begin{array}{c} \theta =\mathrm{abs}(\alpha -\beta ) \end{array}\begin{array}{cc} \tan\theta & =\mathrm{abs}\left(\frac{\tan\alpha -\tan\beta }{1+\tan\alpha \cdot \tan\beta }\right)\\ & =\mathrm{abs}\left(\frac{\frac{\mathrm{abs}(x_{A}-x_{B})}{\mathrm{abs}(y_{A}-y_{B})}-\frac{\mathrm{abs}(x_{B}-x_{C})}{\mathrm{abs}(y_{B}-y_{C})}}{1+\mathrm{abs}\left(\frac{\left(x_{A}-x_{B}\right)}{\left(y_{A}-y_{B}\right)}\cdot \frac{\left(x_{B}-x_{C}\right)}{\left(y_{B}-y_{C}\right)}\right)}\right) \end{array} \end{array},$$

(2) $$\tan\theta =\mathrm{abs}\left(\frac{\frac{\Delta x_{AB}}{\Delta y_{AB}}-\frac{\Delta x_{BC}}{\Delta y_{BC}}}{1+\frac{\Delta x_{AB}}{\Delta y_{AB}}\cdot \frac{\Delta x_{BC}}{\Delta y_{BC}}}\right)\;\mathrm{where}\;\begin{array}{l} \Delta x_{AB}=\mathrm{abs}(x_{A}-x_{B}),\begin{array}{cc} & \Delta y_{AB}= \end{array}\mathrm{abs}(y_{A}-y_{B})\\ \Delta x_{BC}=\mathrm{abs}(x_{B}-x_{C}),\begin{array}{cc} & \Delta y_{BC}= \end{array}\mathrm{abs}(y_{B}-y_{C}) \end{array}$$

(3) $$\tan\theta =\mathrm{abs}\left(\frac{\Delta y_{BC}-\Delta y_{AB}}{1+\Delta y_{AB}\cdot \Delta y_{BC}}\right)\;$$

An issue of turning angle based compression is that the amount of data reduction is affected by noise as noise usually causes local fluctuation, resulting in many local peak points or points with large turning angles. This will cause a large number of undesired points to be recorded and reduce the CR. A low pass filter in the analog frontend can be applied to reduce the high frequency noise to some extent, but it cannot completely remove the local fluctuation. To reduce the impact of the local fluctuation, we propose a window-based turning angle detection method. As shown in Figure 4f, for each point, we will first check whether its previous points or subsequent points move towards the same direction. This is done by checking if the increments (i.e., ∆y) of the most points (e.g., 80%) in an N-point window before or after the checked point have the same signs. If this is met, it indicates that this is unlikely to be local fluctuation caused by noise. Then we start performing the turning angle detection to the checked point to decide whether it is a feature point. The detection window check includes backward window check (for previous N points) and forward window check (for subsequent N points). When either of them meets the condition, the turning angle detection will be triggered. If the point is detected as a feature point, the counter value is reset to 0. With window-based turning angle detection, we can significantly reduce the number of undesired points caused by noise and keep the true feature points.

### 2.2. Adaptive Tuning of Angle Threshold and Window Size

As discussed before, a fixed detection threshold may result in feature loss or low CR due to the variation of signal characteristics from person to person or from channel to channel. To address this issue, we propose to adaptively tune the turning angle threshold according to the computed error between the original waveform and compressed waveform. Here the error between the original signal *X_org_*(*n*) and reconstructed signal *X_rec_*(*n*) is evaluated using percentage root-mean-square difference (PRD), shown in Equation (4), as it is a metric commonly used to evaluate signal distortion in lossy compression methods. Equation (5) shows the definition of CR, where *B_O_* is the number of bits in the original signal, and *B_C_* is the number of bits in the compressed signal, including the feature points and the interval values.

(4) $$PRD(\%)=100\times \sqrt{\frac{\sum_{n=0}^{N}{\left(X_{org}(n)-X_{rec}(n)\right)}^{2}}{\sum_{n=0}^{N}{\left(X_{org}(n)\right)}^{2}}},$$

(5) $$CR(\%)=100\times \frac{B_{O}-B_{C}}{B_{O}},$$

**Figure 5:**
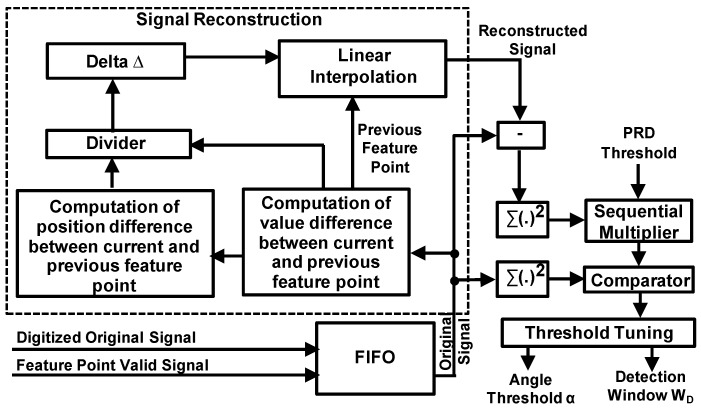
Adaptive threshold tuning module.

Figure 5 illustrates the adaptive threshold tuning module. A FIFO (First-In First-Out) buffer is used to store the original input signal and the extracted feature points over a time period. To minimize the size and power consumption of the FIFO, for the extracted feature points, we only store the one-bit valid signal instead of the values of feature points, as the values are already contained in the original signal. Therefore, for each data in the FIFO, the most significant bit is the one-bit valid signal (indicating whether this data is feature point or not) while the rest is the original data. The reconstructed signal is obtained by linear interpolation using the extracted feature points and interval values. After that, the PRD is computed from the original signal and reconstructed signal, and compared with a preset PRD target. If the computed PRD is less than the PRD target, we will increase the angle threshold to increase CR which will enlarge the PRD, otherwise the angle threshold is reduced to reduce the CR with reduced PRD. In this way, the PRD will finally converge to the preset PRD target while the maximum CR is achieved. In most cases, the variation of signal characteristics is static or slowly changing. Therefore, the adaptive tuning module can be activated infrequently to minimize the power consumption. The activation frequency will depend on the variation frequency of the characteristics of the input signal. For example, when the input signal is an ECG signal, it can be determined according to the profile of user activity. As human activity normally changes slowly (tens of seconds to minutes), the activation frequency can be set to tens of seconds. 

### 2.3. Near-Threshold Design

Sub/near-threshold operation has been proven to be a promising technique to significantly reduce the power consumption in digital circuits by scaling the supply voltage to near/sub-threshold region (around CMOS transistor threshold voltage) when high performance is not required [6,9]. Compared to sub-threshold operation, near-threshold operation has less performance degradation and process variation with only a slight increase in power consumption. In the proposed compression engine, near-threshold design is adopted to reduce the power consumption while meeting the required performance for typical health monitoring applications including ECG, EEG, blood pressure etc., where the sampling frequencies range from Hz up to kHz [6].

To enable near-threshold operation, a standard cell library is pruned by removing the high fan-in cells with more than three inputs as these cells are found to have performance or functionality issues at ultra-low voltages by circuit simulations. After that, the standard cell library is re-characterized at 0.5 V in the near-threshold region. An ultra-low voltage level shifter based on modified Wilson current mirror [26] is used to interface between the ultra-low voltage biomedical compression engine and the I/Os (Input/Output). By scaling the core operating voltage for the nominal voltage at 1.8 V to the near-threshold voltage at 0.5 V, a significant power saving by 5~10x can be achieved for healthcare and biomedical sensor node processor [12,13,17,18].

## 3. Post-Layout Simulation Results and Discussion

**Figure 6:**
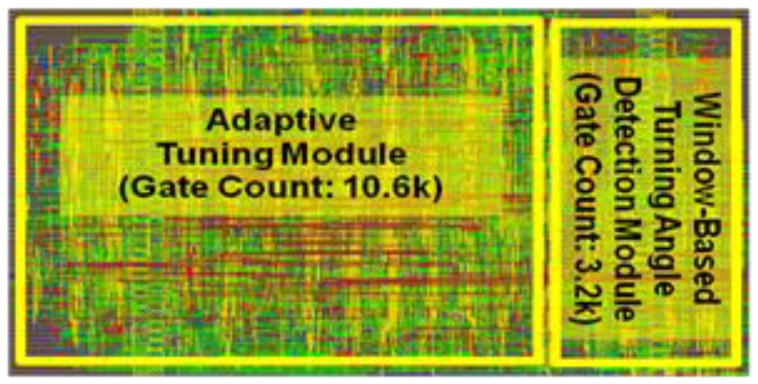
Layout of the proposed biomedical processing engine.

**Figure 7:**
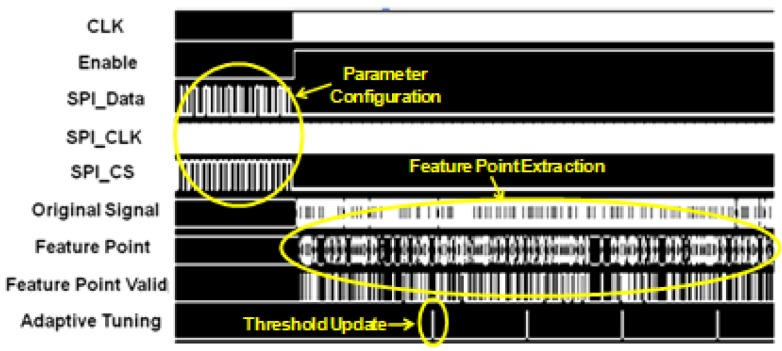
Operation waveform from post-layout simulation.

The proposed design has been implemented in a 0.18 µm CMOS technology. Figure 6 shows the layout of the design. In this study, the proposed biomedical signal compression engine was applied to ECG compression for demonstration. The input is a digitized ECG signal with baseline drift and noise from MIT-BIH arrhythmia database. The output data (i.e., the 16-bit feature point values and the 5-bit interval values) are recorded from post-layout simulation and used for signal reconstruction in MATLAB. Figure 7 shows the operation waveforms captured from the post-layout simulation. After the reset, the parameters of the compression engine—such as angle threshold, target PRD, sampling frequency, and adaptive tuning frequency—are configured through a SPI (serial-to-parallel interface) interface. Then the compression engine is enabled to sample data from external database at 360 Hz. Window-based turning angle detection is performed for feature point extraction. The angle threshold is updated at 200 Hz through adaptive threshold tuning.

**Figure 8:**
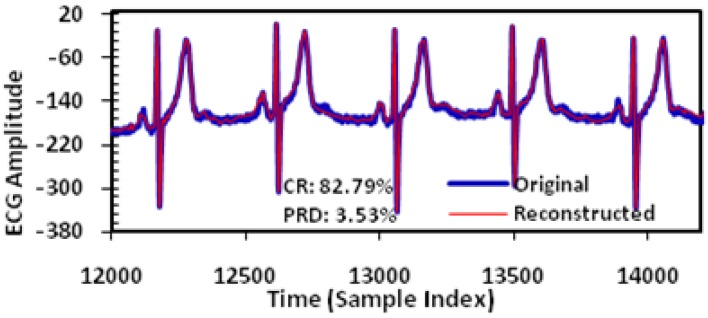
Original and reconstructed ECG signal (No. 117 record).

**Figure 9:**
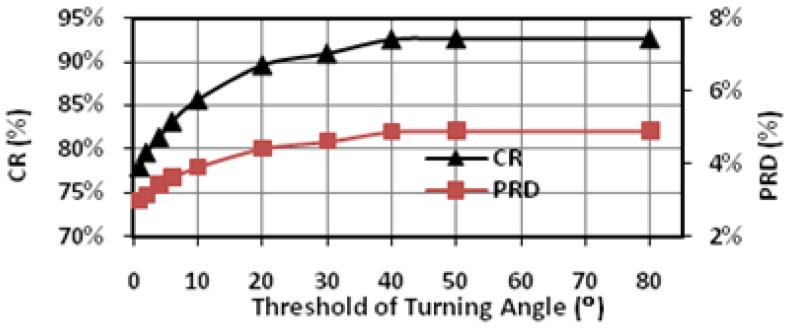
Impact of turning angle threshold on CR and PRD (No. 117 record).

**Figure 10:**
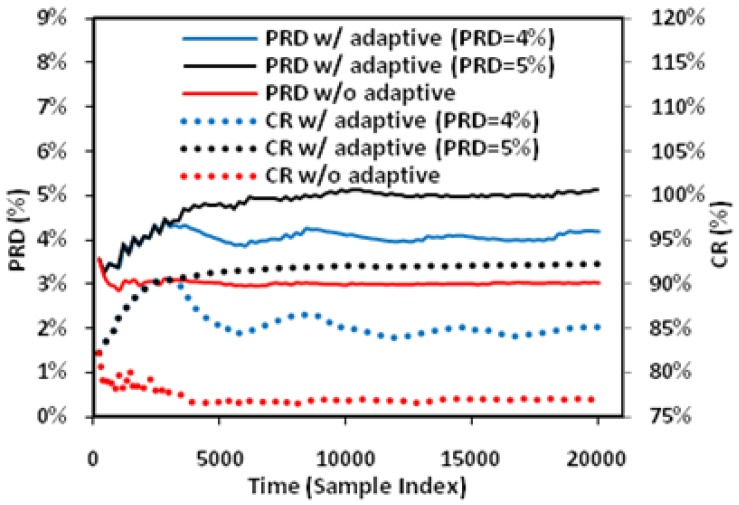
Effect of adaptive angle threshold tuning.

In this study, extensive post-layout simulations have been conducted to evaluate the performance of proposed adaptive biomedical signal compression engine design with ECG waveform (No. 117 record) from MIT-BIH arrhythmia database. The compressed ECG output data have been captured from the simulation and fed to MATLAB for post-processing. Firstly, a reconstructed signal with small PRD (3.53%) and large CR (82.79%) can be achieved with fixed angel threshold (Figure 8); secondly, impact of turning angle threshold on CR and PRD has been studied to show that tuning the angle threshold is able to increase the CR while not compromising the PRD much (Figure 9); third, simulation has shown that the proposed adaptive turning angle threshold can automatically achieve maximum CR to 93% from 78%, while meeting the required signal quality specified by a PRD at 5% (Figure 10); finally, the proposed adaptive biomedical compression engine has also been compared with state-of-the-art designs in terms of compression performance and energy efficiency. Simulation results show that the proposed technique has achieved the best PRD result with similar CR among several existing lossy compression engines, while consuming the lowest power at 39 nW (Table 1). With such low power consumption, the proposed design can extend the lifetime of a coin battery (Energizer CR2032) by more than 10 times against the state-of-the-art compression engine. The detailed analysis and discussion of the simulation results are provided as below.

We take the dataset 117 from MIT-BIH ECG database (commonly used for ECG signal compression research) as an example, the original and the reconstructed waveforms of the ECG signal are shown in Figure 8. Here the adaptive threshold tuning is disabled. For the No. 117 record, a CR of 82.79% is achieved which will lead to proportional power reduction in the wireless transmission. The PRD achieved is 3.53% which is classified as ‘good’ or ‘very good’ according to previous studies [27,28,29].

Figure 9 shows the impact of the angle threshold α on the CR and PRD. In this case, we set the window size N to 10. The angle detection is triggered when there are more than 8 points having the increments with the same direction in either the backward or the forward 10-point window. This actually determines the lower bound of CR, because the detection window will limit the number of recorded feature points even without angle detection. As the angle threshold is increased from 0° to 50°, the CR increases from 78% to 93% and the PRD increases from 3% to around 5%. From 50° to 80°, the CR and PRD remain almost constant, indicating that there are very few turning angles within the range in this case. This highlights the importance of adaptive threshold tuning, which is able to adjust the CR according to the variation of signal characteristics of the input signal.

Figure 10 shows the effect of adaptive angle threshold tuning. For a fixed angle threshold of 5° without adaptive tuning, the instant PRD and CR—which are computed based on the historical samples—vary over time at the beginning and settles to around 3% and 77% after around 5000 samples. With adaptive threshold tuning, the angle threshold is automatically adjusted to force the PRD to converge to the preset target values. As a result, the maximum CR is obtained. In the two cases illustrated, the CR is increased from 77% to 86%/93% for achieving the required PRD of 4%/5%. Compared with fixed angle threshold, the proposed method is useful in dealing with variation of signal characteristics to achieve maximum CR while meeting the required signal quality.

To investigate the power consumption, we have performed power analysis using the circuit switching activity extracted from the post-layout simulation. The power Figure together with other parameters is shown in Table 1 and compared with several state-of-the-art designs. The proposed design achieves an average CR of 71.08% and PRD of 5.87% from the 48 records of the MIT-BIH arrhythmia database. For a similar CR, the PRD of the proposed compression engine is the best among the compared lossy compression engines. The power consumption of the proposed compression engine is only 39 nW when performing compression on one-lead 260-Hz 12-bit ECG signal at 0.5 V, which is significantly lower than other designs. It is noted for the other designs only the power consumption of compression is used for comparison.

In this study, a typical lithium coin battery, i.e., Energizer 240-mAh CR2032 battery [30], is selected for the power consumption comparison of the proposed design with several existing designs in [17,18,25,29]. For a smart ECG sensor node, usually a 20% of battery power can be allocated to the digital signal processor while the rest is allocated to wireless transceiver, analog front end, power management, and sensor. Furthermore, a 10% of the battery power (i.e., 24-mAh) can be allocated to the data compression module in the digital signal processor. For the proposed 0.5-V adaptive biomedical compression engine, the lifetime of the battery is approximately 36 months to performing 12-lead ECG data compression at 936 nA. The battery life is about 3.2 months and 2.6 months, for the state-of-the-art ultra-low voltage ECG compression engines in [17,18], respectively. For those mini-Watt FPGA/DSP solutions [25,29] without any ultra-low-power design technique, the battery life is less than half day. Considering a wireless transceiver with energy consumption of 6 nJ/bit [31], the energy consumption for transmitting a 256-sample ECG segment after compression by our proposed compression engine will be ~17.5 µJ only. Therefore, 24-hour healthcare data collection from a 12-lead 360-Hz 12-bit ECG recording system generates about 522 MBytes raw data for real-time transmission to the remote base station or cloud sides. With the proposed ultra-low-power compression engine, the energy consumption required by the wireless transceiver is approximately 7.42 mJ, which achieves a significant savings of about 71% from the energy consumption without any data compression.

## 4. Conclusions

In this paper, we propose a turning angle based biomedical signal compression engine with adaptive threshold tuning and near-threshold operation. The proposed design has extremely low power consumption while achieving large CR and good PRD. Adaptive threshold tuning is proposed to address the variation of signal characteristics from person to person or from channel to channel. In this study, the method is applied to ECG compression, and it achieves an average CR of 71.08% and PRD of 5.87% while consuming only 39 nW, which is significantly lower than several state-of-the-art ECG compression engines with similar CR and PRD. The proposed biomedical signal processing engine is suitable for long-term wearable miniaturized sensor systems to sense and collect healthcare data for remote data analysis in the era of big data and sensor intelligence.

## Figures and Tables

**Table 1:** Comparison of proposed design with other designs.

-	This Work	[17]	[18]	[25]	[29]
Compression Type	Lossy	Lossy	Lossless	Lossy	Lossy
Process Technology	0.18 µm	0.18 µm	0.35 μm	N/A ^1^	N/A ^1^
Sampling Rate	360 Hz	250/500 Hz	512 Hz	250 Hz	360 Hz
Average CR	71.08%	37.5%	60.7%	60%	74.8%
PRD	5.87%	N/R ^2^	N/A ^1^	7.32%	9%
Power Consumption of Compression	39 nW (ASIC)	435 nW (ASIC)	535 nW (ASIC)	~11 mW (FPGA)	2.18 mW (DSP)
Adaptive Threshold Tuning	Yes	No	No	No	No
Gate Count (Gates)	Detection: 3.2 k Adaptive Tuning: 10.6 k	115 k	0.56 k	N/A ^1^	N/A ^1^

^1^ N/A: Not applicable. ^2^ N/R: Not reported.
